# Albuminemia as a Potential Predictor of Clinical Outcomes in Patients with Severe Traumatic Brain Injury (TBI)

**DOI:** 10.3390/jcm14217499

**Published:** 2025-10-23

**Authors:** Luka Stepanovic, Usha Govindarajulu, George Agriantonis, Navin D. Bhatia, Jasmine Dave, Shalini Arora, Zahra Shafaee, Kate Twelker, Jennifer Whittington, Bharti Sharma

**Affiliations:** 1 Trauma Unit, Department of Surgery, NYC Health and Hospitals/Elmhurst Hospital Center, Elmhurst, NY 11373, USA; lukastepanovic01@gmail.com (L.S.); agriantg@nychhc.org (G.A.); bhatian1@nychhc.org (N.D.B.); davej@nychhc.org (J.D.); arorash@nychhc.org (S.A.); shafaeez1@nychhc.org (Z.S.); harrisj20@nychhc.org (J.W.); 2 Trauma Unit, Department of Surgery, Icahn School of Medicine at Mount Sinai, New York, NY 10029, USA; 3 Center of Biostatistics, Department of Population Health Science and Policy, Icahn School of Medicine at Mount Sinai, New York, NY 10029, USA; usha.govindarajulu@mountsinai.org

**Keywords:** albumin level, hypoalbuminemia, traumatic brain injury, injury severity score, severe trauma

## Abstract

**Objectives:** Albumin levels (ALBs) influence clinical outcomes in severe traumatic brain injury (TBI). This study investigates the relationship between ALBs and clinical outcomes to improve prognosis and guide management. **Method:** This is a single-center, retrospective study of patients with severe TBI from 1 January 2020 to 31 December 2023. ALBs were measured at the following times: Hospital admission (TA), ICU admission (RL1), ICU discharge (RL2), hospital discharge (HD), and patient death (PD) if applicable. Patient descriptors and clinical outcomes such as gender, age, race, hospital length of stay (HLOS), ICU length of stay (ICU LOS), ventilation days (VD), the Glasgow Coma Scale (GCS), the Injury Severity Score (ISS), and mortality were assessed. **Results:** ALBs were grouped into the following categories: extreme hyperalbuminemia (≥5.5 g/dL), hyperalbuminemia (4.7–5.4 g/dL), normal albuminemia (3.5–4.7 g/dL), hypoalbuminemia (2.5–3.5 g/dL), and extreme hypoalbuminemia (<2.5 g/dL). Among 925 severe-TBI patients (76% male; mean age = 53 y), admission albumin was normal (3.5–4.7 g dL^−1^) in 65.0%, hypoalbuminemic (<3.5 g dL^−1^) in 25.2%, and hyperalbuminemic (>4.7 g dL^−1^) in 9.4%. By ICU discharge, albumin shifted upward: extreme hyperalbuminemia (≥5.5 g dL^−1^) 62.6%, hyperalbuminemia 15.8%, normoalbuminemia 20.4%, and hypoalbuminemia ≤ 1.3%. In-hospital mortality was 12.7% (117/925) and did not vary by either the admission or discharge albumin category (χ^2^ = 3.47, *p* = 0.32). The median hospital length of stay was 5 d (IQR ≈ 11 d). ICU stay was 1.3 d, and ventilator use was 0 d; none differed significantly across albumin strata (all Kruskal–Wallis *p* > 0.10). **Conclusions:** Although serum ALBs changed substantially during acute care with shifting from frequent hypoalbuminemia on admission to predominant hyperalbuminemia at ICU discharge, albumin concentration was not independently associated with mortality or resource utilization. In modern neuro-critical practice, where protein deficits are rapidly corrected, albumin serves mainly as a therapeutic target rather than a stand-alone prognostic marker in severe TBI.

## 1. Introduction

Traumatic brain injury (TBI) represents a major global health challenge and is a leading cause of injury-related death and disability worldwide [1]. Each year, TBI affects tens of millions of people, with some estimates suggesting around 69 million new cases annually (other analyses projecting 50–60 million when accounting for mild injuries) and imposes enormous economic burdens [2]. According to the CDC, in the United States alone, TBI contributed to over 69,000 deaths in 2021, and the annual healthcare costs for TBI are estimated in the tens of billions of dollars [3]. Beyond the immediate fatalities, many survivors suffer long-term neurological deficits, making TBI a significant source of lifelong disability. Severe TBI triggers a complex cascade of secondary injuries and systemic complications that extend well beyond the primary brain insult. In the acute phase, patients can develop cerebral ischemia, edema, elevated intracranial pressure, hydrocephalus, and other intracranial sequelae [3]. TBI also initiates a robust systemic inflammatory and neuroendocrine response that can disrupt the function of multiple organ systems. Consequently, extracranial complications are common, including coagulopathies, cardiovascular instability, acute lung injury, renal dysfunction, and other components of multiorgan failure. Improving early recognition and management of these secondary insults is essential for enhancing TBI outcomes.

Among the systemic disturbances observed after trauma and TBI, derangements in serum protein homeostasis, particularly hypoalbuminemia (low albumin levels) of total serum protein content, account for approximately 80% of the plasma oncotic pressure [4,5]. Synthesized by the liver at approximately 16 g per day in healthy adults, albumin plays a critical role in maintaining intravascular volume (colloid osmotic pressure) and serves as a carrier for hormones, drugs, and other molecules. Trauma and TBI provoke an acute inflammatory response in which albumin behaves as a negative acute-phase reactant. Inflammation and stress hormones suppress hepatic albumin synthesis and increase capillary permeability, leading to the loss of albumin into the interstitial space [6]. Hemodilution from aggressive fluid resuscitation and hemorrhage can further lower serum albumin concentrations [7]. As a result, hypoalbuminemia is frequently observed in critically ill and injured patients. A significant proportion of severe TBI patients present with or develop hypoalbuminemia shortly after injury [7]. In general, hospitalized populations, more than half of patients may have low albumin by the time of admission or discharge [6], and trauma patients are no exception. One recent study reported that 53.1% of adult trauma patients exhibited early-onset hypoalbuminemia (albumin < 3.5 g/dL) within the first week after injury [8]. Similarly, among patients with severe TBI, roughly 30–40% are hypoalbuminemic on presentation [9], reflecting the combined effects of pre-injury health status, blood loss, and the acute-phase reaction to trauma. Low ALBs are strongly associated with increased morbidity and mortality in both acute and chronic disease states [5].

It is important to note, however, that ALBs can also be artificially inflated by clinical interventions such as aggressive fluid resuscitation, depending on the supplementation and solutions used. This therapeutic effect interferes with the ability to determine the relationship between albumin and intrinsic disease severity, possibly obscuring ALB’s additional clinical relevance as a prognostic biomarker. In spite of this, due to its critical role as an acute biomarker, any potential prognostic value should be evaluated.

Given these implications, there is growing interest in using albumin-based metrics as prognostic tools in TBI. Despite the extensive documentation of hypoalbuminemia’s prevalence and prognostic associations, relatively little is known about how serum ALBs evolve throughout hospitalization after severe TBI and how these longitudinal patterns relate to patient outcomes. Most prior studies have assessed albumin at a single time point (e.g., on admission). It remains unclear whether persistent or worsening hypoalbuminemia during hospital stay confers additional risk or if early correction of low albumin improves outcomes in TBI. Standard definitions of hypoalbuminemia also vary across the literature. Normal adult serum albumin is typically 3.5–5.0 g/dL, and levels below ~3.5 g/dL are considered low [5]. Severe or “extreme” hypoalbuminemia is sometimes defined at a stricter cutoff (e.g., <2.5 g/dL) to denote critically low protein reserves. In our analysis, we classified ALBs into five categories for granular assessment: extreme hypoalbuminemia (<2.5 g/dL), hypoalbuminemia (2.5–3.4 g/dL), normoalbuminemia (3.5–4.6 g/dL), hyperalbuminemia (4.7–5.4 g/dL), and extreme hyperalbuminemia (≥5.5 g/dL) [10,11,12]. Using these definitions, we aimed to characterize the trajectory of albumin changes in patients with severe TBI and to determine the relationship between albumin abnormalities (at admission and over time) and clinical outcomes.

## 2. Method

### 2.1. Study Population

This was a single-center retrospective study conducted at a level 1 trauma center in Queens, New York City, NY, USA, verified by the American College of Surgeons. We included all patients who presented with severe TBI between 1 January 2020 and 31 December 2023. Inclusion criteria were an Abbreviated Injury Severity (AIS) score of 3 or higher and available ALB measurements for patients who were either discharged or transferred. Patients were excluded if they had a COVID-19 infection at the time of injury, died or were discharged within 24 h of admission, or had an AIS score below 3 (representing minor or moderate injuries).

In total, we identified 1040 patients with severe TBI, and 925 patients met the final inclusion criteria for the study. The research protocol was approved by the institutional review board (IRB) at Elmhurst Hospital Center (IRB #24-12-092-05G). Data for all eligible patients were obtained from the National Trauma Registry of the American College of Surgeons (NTRACS) database at Elmhurst Hospital Center. NTRACS catalogs various types of TBIs, but for clarity, only combinations relevant to this study were included in our analysis. When necessary, individual patient charts were reviewed to gather all additional information required for the research.

Patients were identified by mechanism of injury, cause, primary mechanism, ICD9 or ICD10 E-code, and AIS (head region). The AIS score ranges from 1 to 6 for each body region. Severe TBI was defined as a Glasgow Coma Scale (GCS) of 8 or less after resuscitation but before administration of sedation. Standard Advanced Trauma Life Support (ATLS) protocols at our center dictated routine endotracheal intubation for patients with GCS ≤ 8, as well as for those with compromised airways, persistent hypoxia, or severe facial trauma. Intracranial pressure (ICP) monitoring was performed at the discretion of the consulting neurosurgeon, and surgical interventions such as craniotomy or decompressive craniectomy were undertaken when indicated based on imaging and neurosurgical assessment.

ALBs were classified into five categories: Extreme hyperalbuminemia (≥5.5 g/dL), hyperalbuminemia (4.7–5.4 g/dL), normal albuminemia (3.5–4.7 g/dL), hypoalbuminemia (2.5–3.5 g/dL), and extreme hypoalbuminemia (<2.5 g/dL). We examined potential statistically significant differences in ALB measured at multiple time points: Hospital admission (TA), ICU admission (RL1), ICU discharge (RL2), hospital discharge (HD), and patient death (PD) if applicable. The clinical outcomes analyzed included emergency department, ICU, and hospital length of stay, duration of mechanical ventilation, and mortality.

### 2.2. Data Collection

Data was collected using a spreadsheet (Excel) as a data collection tool, integrating all relevant data elements such as patient demographics, clinical outcomes, and biochemical measurements. Baseline admission information included demographic variables (age, sex, race, and ethnicity), AIS, and ICD-coded injury descriptions. Age was categorized into groups: pediatric (<15 years), young adults (15–25 years), older adults (24–64 years), and elderly (>65 years). Sex was classified as male or female. Additionally, we documented the mechanism of injury as either blunt or penetrating.

### 2.3. Statistical Analysis

Pearson Chi-square tests were conducted to test the association between certain categorical variables. The Kruskal–Wallis test was used to test if various scores and lengths of stays differed between different ALBs for ALB measured at different points of stay. The analyses were conducted in SAS Version 9.4. A significance level of 0.05 was used for all analyses.

## 3. Results

Patient Cohort Characteristics: A total of 925 patients with severe TBI met the inclusion criteria for this analysis. The cohort was predominantly male (706 men, 76.3%; 219 women, 23.7%). The average age was 53 years, with 1.8% of patients under 15 years, 6.2% aged 15–23, 60.3% aged 24–64, and 31.7% aged 65 or older. Mechanism of injury was overwhelmingly blunt trauma (906 patients, 98%), with only 19 penetrating injuries. In-hospital mortality was 12.7% (117 deaths), and 87.3% (808 patients) survived until discharge. Baseline demographics and clinical characteristics are summarized in Table 1.

**Figure 1:**
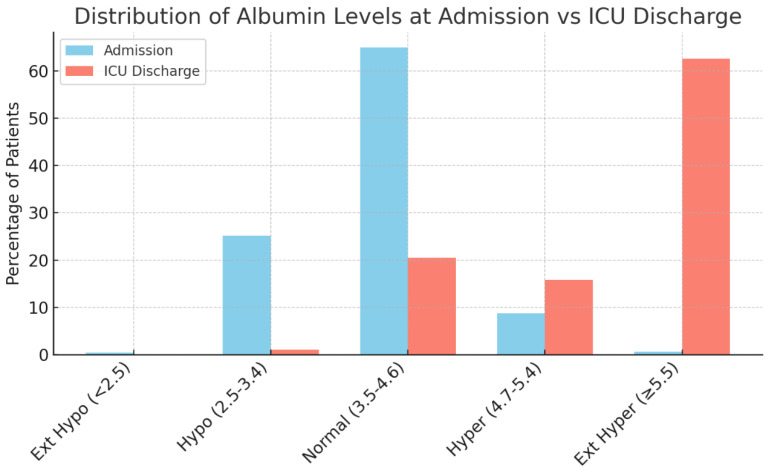
Distribution of serum ALBs at hospital admission versus ICU discharge. At admission, the majority of patients had normal albumin (3.5–4.6 g/dL, blue bars), and about one-quarter were hypoalbuminemic (2.5–3.4 g/dL). By ICU discharge, ALBs shifted upward: over 60% of patients had extreme hyperalbuminemia (≥5.5 g/dL, red bars), and the prevalence of hypoalbuminemia fell dramatically. (Ext Hypo = extreme hypoalbuminemia; Ext Hyper = extreme hyperalbuminemia).

Serum Albumin Distribution: At hospital admission, albumin was in the normal range for the majority of patients (601/925, 65.0%). Hypoalbuminemia was present in about one-quarter of the cohort (233 patients, 25.2%), whereas hyperalbuminemia was less common (81 patients, 8.8%). Extreme deviations were rare on admission; only 4 patients (0.43%) had albumin < 2.5 g/dL, and 6 patients (0.65%) had albumin ≥ 5.5 g/dL. By ICU discharge, the albumin distribution had shifted markedly (Figure 1). Table 2 details the frequencies at these time points. The vast majority of patients achieved hyperalbuminemia or extreme hyperalbuminemia by the time of ICU transfer/discharge. Specifically, extreme hyperalbuminemia (≥5.5 g/dL) became the most prevalent category, observed in 580 patients (≈62.7% of those with ICU-discharge labs). An additional 15.8% (147 patients) had albumin in the 4.7–5.4 g/dL range. Thus, nearly four in five patients were hyperalbuminemic at ICU discharge. The proportion of normoalbuminemia dropped to ~20% (189 patients), and very few patients remained hypoalbuminemic (only 10 patients, ~1%). Notably, no patients had extreme hypoalbuminemia by ICU discharge. This represents a significant shift from admission, where hypoalbuminemia was common and severe hyperalbuminemia was essentially absent. The mean length of hospital stay in this cohort was 12 days, with a mean ICU stay of 4 days and an average of 2 days on mechanical ventilation (ventilator days).

Demographic characteristics were also examined with the application of the ALB groupings. When the hospital admission ALBs of different age groups were analyzed, it was found that extreme deviations were rare across all ages (Table 3). However, hypoalbuminemia on admission did cluster towards middle-aged (24–64 y) and elderly (≥65 y) patients. No further significance was noted. When ALBs between female and male patients were examined, distributions were found to be similar and therefore not statistically significant (Table 4).

Other variables, including the mechanism of injury (Table 5) and AIS-Head (Table 6), were also looked at to discern any clinically significant variations in each ALB group. There were no meaningful differences in the groupings of either variable that could contribute to or disprove prognostic potential.

Clinical Outcome Prediction Potential: We next analyzed clinical outcomes across the albumin categories. Key outcomes, including in-hospital mortality, hospital length of stay (HLOS), ICU length of stay (ICU LOS), and days on mechanical ventilation, were compared between albumin groups at hospital admission and ICU discharge. Overall, no statistically significant differences in these outcomes were observed across the albumin categories. In-hospital mortality rates were similar regardless of hospital admission (Table 7). ALB: for instance, patients with hypoalbuminemia on admission had a mortality of 14.2% versus 12.2% in normoalbuminemic patients, and this difference was not significant (*p* = 0.71). Patients presenting with extreme hyperalbuminemia on hospital admission (n = 6) all survived, but this group was too small for meaningful comparison. By ICU discharge, albumin category still showed no association with survival, approximately 13.5% of patients in each major albumin group had died (e.g., 13.4% of those with ≥5.5 g/dL vs. 13.2% with 3.5–4.6 g/dL), and the distribution of ALBs did not differ significantly between survivors and non-survivors (χ^2^ = 3.47, *p* = 0.324).

Other outcome measures demonstrated a similar lack of significant variation. Hospital LOS did not differ appreciably among the five albumin categories. For example, the median HLOS was on the order of 7–8 days in each group, and a nonparametric comparison found no significant variance (Kruskal–Wallis test, *p* > 0.5). ICU LOS and ventilator days were likewise comparable across albumin strata. Patients with low admission albumin (indicating possible malnutrition or severity) did not have significantly longer stays than those with normal or high albumin, nor did patients with extreme hyperalbuminemia exhibit shorter stays. Statistical tests confirmed the absence of strong associations; for instance, albumin at ICU discharge had no significant relationship with total hospital days or days in intensive care (*p* > 0.1 for both comparisons).

Overall, these results indicate that ALB alone was not a clear predictor of clinical outcomes in this cohort. Unlike other laboratory parameters studied in TBI (e.g., potassium levels), albumin imbalances showed no significant influence on mortality or length of stay in our analysis. Patients in all albumin categories had broadly similar outcomes. The most striking finding was the dynamic change in ALBs throughout hospitalization: whereas hypoalbuminemia was common on admission, interventions such as aggressive volume/albumin management or hemoconcentration in the ICU presumably led to a high prevalence of hyperalbuminemia by ICU discharge (Table 2, Figure 1). Importantly, this rise in albumin did not translate into divergent outcome trajectories between groups. Chi-square analyses and Kruskal–Wallis tests yielded no significant differences in mortality, discharge disposition, or durations of care when comparing the albumin categories (all *p*-values non-significant, generally *p* > 0.3 for categorical outcomes and *p* > 0.1 for continuous outcomes). Thus, in this retrospective cohort of severe TBI patients, serum ALBs, despite varying widely between admission and ICU discharge, were not significantly associated with clinical outcomes.

## 4. Discussion

### 4.1. Key Findings

This single-center retrospective study of 925 severe TBI patients found that serum ALBs underwent a profound shift during hospitalization, yet showed no significant association with key clinical outcomes. On admission, about two-thirds of patients had normal albumin (3.5–4.6 g/dL), and roughly one-quarter presented with hypoalbuminemia (<3.5 g/dL). Extreme deviations were very uncommon initially (<1% for albumin <2.5 or ≥5.5 g/dL). By ICU discharge, however, ALBs had risen dramatically; nearly 80% of patients became hyperalbuminemic (>4.7 g/dL), with over 60% reaching extreme hyperalbuminemia (≥5.5 g/dL). This striking upward shift suggests that aggressive volume resuscitation and/or albumin supplementation in the ICU corrected initial deficits, virtually eliminating severe hypoalbuminemia by the time of transfer out of intensive care. While this therapeutic intervention could be considered an issue in evaluating ALB, these values are standardized through resuscitation and intervention protocols. Their corresponding clinical outcomes are compared not only against each other, but also against the findings of other literature done with different fluctuations in ALBs.

Despite these wide fluctuations in albumin, no statistically significant differences in outcomes were observed across albumin categories. In-hospital mortality was similar regardless of admission ALB: patients with hypoalbuminemia on arrival had a mortality of ~14.2% versus 12.2% in normoalbuminemic patients (*p* = 0.71). Even patients with extreme hyperalbuminemia at admission (n = 6) all survived, though this group was too small for meaningful comparison. By ICU discharge, survival rates remained comparable across ALBs (~13% mortality in each group), and the distribution of albumin did not significantly differ between survivors and non-survivors (χ^2^ = 3.47, *p* = 0.324). Similarly, other outcome measures showed no notable variation. Hospital length of stay (HLOS) was on the order of 7–8 days for each albumin group, with no significant differences by Kruskal–Wallis test (*p* > 0.5). ICU length of stay and days on mechanical ventilation were likewise equivalent across albumin strata. Patients presenting with low albumin did not experience longer hospitalizations or ICU stays than those with normal or high albumin, nor did high-albumin patients have shorter stays. Statistical analyses consistently confirmed the absence of strong associations; all *p*-values were well above significance thresholds for both categorical outcomes (mortality, discharge disposition) and continuous outcomes (durations of care).

Overall, the key finding is that serum ALB per se was not a clear prognostic predictor in this cohort of severe TBI patients. This contrasts with some other TBI-related laboratory parameters (e.g., dysnatremia or dyskalemia) that have shown prognostic significance. In our analysis, patients in all albumin categories had broadly similar clinical outcomes. The most notable observation was the dynamic albumin change over time: initial hypoalbuminemia was common, but after aggressive care in the ICU, most patients became hyperalbuminemic. Crucially, this rise in albumin did not translate into any outcome divergence between groups. In summary, while ALBs varied widely from admission to ICU discharge, these variations did not significantly influence mortality, length of stay, or ventilator days in our severe TBI population.

### 4.2. Comparison to Existing Literature

Our findings can be contextualized against a growing body of literature on albumin in critical illness and TBI. Hypoalbuminemia is widely recognized as a marker of illness severity and has been extremely common in trauma cohorts. For instance, a recent prospective study of 386 trauma patients reported early-onset hypoalbuminemia (albumin < 3.5 g/dL) in 53.1% of cases within the first week [8]. Similarly, among severe TBI populations, roughly 30–45% of patients have been found to present with hypoalbuminemia on admission [13,14]. This prevalence aligns with our observation of ~25% hypoalbuminemia at admission (noting our cohort’s exclusion of moribund 24-h survivors might lower this rate). Historically, low albumin has often been associated with worse outcomes. Nayak et al. (2020), studying 80 severe head injury patients, found that those with admission albumin < 3.5 g/dL had significantly poorer 6-month neurological outcomes compared to normoalbuminemic patients (72.5% vs. 90% good recovery; *p* = 0.01) [15]. In their analysis, hypoalbuminemia emerged as an independent predictor of unfavorable outcome (adjusted odds ratio ~6.3 for poor Glasgow Outcome Score). This stands in contrast to our larger cohort, which did not confirm any outcome disadvantage for low-albumin patients in the acute hospital phase. One possible reason is that prior studies often examined longer-term functional outcomes (e.g., 3–6 month GOS) or were limited to smaller sample sizes, whereas our study focused on in-hospital metrics with a robust sample and aggressive albumin correction practices [16,17,18]. It is conceivable that albumin’s impact might manifest more in long-term recovery or untreated states, whereas prompt critical care interventions (as in our cohort) can mitigate short-term risks associated with low albumin.

Recent research has also explored composite markers that integrate albumin with other indicators of physiologic stress. These studies generally reinforce the notion that while albumin alone has little prognostic value, it may be a stronger predictor when considered in combination with markers of inflammation or perfusion. For example, the C-reactive protein/albumin ratio (CAR), an index of systemic inflammation and nutritional status, has shown promise in TBI prognostication [19]. Wang et al. (2023) reported that TBI patients who did not survive had a markedly higher admission CAR than survivors (median ~3.8 vs. 2.6, *p* < 0.001) [20]. In multivariate analysis, CAR was an independent risk factor for in-hospital mortality, outperforming some conventional predictors. This mirrors findings in other conditions that an elevated CAR portends worse outcomes, likely because it captures the dual hit of inflammation (high CRP) and poor protein reserves (low albumin). Similarly, the lactate-to-albumin ratio (LAR) has been investigated as a prognostic tool in neurotrauma. Lactate reflects tissue hypoperfusion and metabolic stress, while albumin reflects nutritional and inflammatory status; a high LAR thus indicates simultaneous shock and hypoalbuminemia. Lee et al. (2023) found that an elevated LAR on admission was associated with significantly higher 24-h mortality in TBI (odds ratio ~2.0 per unit increase in LAR) [21]. Correspondingly, Wang et al. (2022) observed in 273 TBI patients that non-survivors had a mean LAR roughly double that of survivors (1.09 vs. 0.53) and that LAR was an independent predictor of in-hospital death (OR 1.70, *p* = 0.022) [22]. Notably, in that study, albumin by itself did have some prognostic signal—admission albumin under 3.5 g/dL was associated with worse outcomes—but the combined LAR provided better discrimination (area under ROC 0.78 for LAR vs. 0.74 for albumin alone). These findings suggest that albumin contributes meaningfully to outcome prediction when interpreted in context with concurrent physiologic disturbances. Our results, which showed no outcome difference across albumin categories alone, are thus consistent with the idea that albumin as a solitary measure may be an insufficient prognostic discriminator, even if extreme hypoalbuminemia indicates illness severity. Large critical-care studies have noted that while hypoalbuminemia independently correlates with higher mortality, its predictive power is limited. For example, an analysis of >18,000 ICU patients found that serum albumin < 3.0 g/dL was associated with ~1.5-fold higher odds of hospital death, but albumin had low sensitivity and specificity for mortality (many nonsurvivors had normal albumin and vice versa) [23]. This aligns with our finding that albumin categories did not cleanly stratify outcomes; other factors in TBI likely dominate prognosis unless albumin is exceedingly low.

Inflammation-based albumin indices further underscore albumin’s contextual role. Aside from CAR and LAR, the neutrophil-to-albumin ratio (NAR) has recently been proposed as a superior composite biomarker in TBI [24,25]. In a recent study, they showed that NAR had stronger prognostic performance than traditional measures like neutrophil–lymphocyte ratio [26]. Patients with poor 3-month neurological outcomes had significantly higher NAR on admission than those with good recoveries. Importantly, that study noted that hypoalbuminemia on its own was linked to elevated 30-day mortality rates in TBI patients, reinforcing the general association between low albumin and adverse outcome, but also that coupling albumin with an inflammatory marker like neutrophil count improved predictive accuracy. In summary, the literature suggests that albumin derangements after TBI are common and generally correlate with injury severity and outcome, but in isolation, albumin is a blunt prognostic instrument. Our findings add to this context by demonstrating in a large cohort that ALB, when aggressively managed, did not differentiate patient trajectories. This contrasts with smaller studies that reported significant outcome differences with hypoalbuminemia in non-traumatic care, as well as with composite indices where albumin plays a part [27,28]. One notable difference in our cohort is the routine correction of hypoalbuminemia; by ICU discharge, most patients were hyperalbuminemic, a scenario seldom reported elsewhere. Thus, our results support the notion from broader critical care research that while hypoalbuminemia is a red flag biologically, its prognostic impact can be blunted by prompt, effective supportive care. Indeed, the role of albumin in TBI is still debated on the therapeutic front: earlier trials (SAFE-TBI) suggested that infusing hypo-oncotic albumin (4% solution) in TBI resuscitation was associated with worse outcomes, whereas a more recent meta-analysis indicated that using hyperoncotic 20–25% albumin (as part of the “Lund concept” for intracranial pressure management) was associated with lower mortality (14.5% vs. 38.1% in controls, *p* = 0.002) [7]. These disparate findings reflect that albumin’s effects may depend on context, such as concentration, fluid shifts, and timing, and highlight the complexity in comparing across studies. In the context of prognostication, our study’s null results align with those larger patterns: albumin is a valuable clinical indicator of physiologic stress, but not a stand-alone predictor of outcome unless combined with other factors or left uncorrected.

### 4.3. Implications of Study Findings

From a clinical perspective, our findings suggest that routine albumin measurements in severe TBI may be more useful for guiding supportive therapy than for predicting patient outcomes. The dramatic rise in ALBs observed during ICU care indicates that modern critical care interventions (e.g., intravenous albumin administration, concentrated feeding, or hemoconcentration via diuresis) can effectively reverse initial hypoalbuminemia. Consequently, any potential prognostic disadvantage of low albumin on admission appears to have been mitigated in our cohort. In practical terms, this means that an admission albumin level below normal should prompt clinicians to ensure adequate volume resuscitation and nutritional support, but it should not be viewed as a definitive harbinger of poor outcome in isolation. Traditional teaching has linked hypoalbuminemia to higher morbidity and mortality, yet our data indicate that when hypoalbuminemia is promptly corrected, severe TBI patients can achieve outcomes comparable to those with normal albumin. This underscores the importance of addressing reversible factors (like protein depletion) early in the ICU course.

It is also notable that ALBs by ICU discharge were universally high without corresponding improvements in outcome. This implies there is no clear benefit (nor overt harm) in driving albumin to supranormal ranges with aggressive replacement. Therefore, management protocols might prioritize maintaining adequate albumin for colloid osmotic support and medication binding, but ultra-high ALBs should not be expected to confer additional outcome advantages. Our study reinforces that neurologic injury severity and systemic complications (rather than albumin per se) remain the dominant outcome determinants in severe TBI. Going forward, clinicians should continue to treat albumin derangements as an addressable marker of physiological stress, but not rely on albumin alone for prognostication. Instead, a more holistic approach, such as integrating albumin with other indicators (e.g., lactate, CRP, immune markers), may better inform risk, as suggested by emerging composite scores. In summary, our findings imply that maintaining albumin within a normal range is an important supportive goal in severe TBI care, but beyond that, ALB itself provides limited prognostic information, especially in the context of proactive critical care management.

### 4.4. Strengths, Limitations, and Future Perspectives

This investigation offers several strengths that add confidence to its principal finding, which is that serum albumin, when aggressively managed, does not independently predict acute outcomes in severe TBI. First, the cohort is large (n = 925) for a single-center neuro-trauma study and encompasses consecutive admissions over four years, reducing selection bias. Second, albumin was measured at multiple predefined junctures (admission, ICU admission, ICU discharge, hospital discharge, and death), permitting a rare view of its trajectory across the entire acute-care episode rather than the single time-point snapshots typical of earlier reports. Third, the institution’s standardized resuscitation, nutrition, and neurosurgical protocols minimize inter-patient treatment variability, allowing clearer attribution of observed albumin shifts to routine critical-care interventions. Finally, outcomes were captured comprehensively, including mortality, hospital and ICU length of stay, and ventilator days, providing a multidimensional assessment of clinical course.

Important limitations temper these strengths. The retrospective design precludes firm causal inference and is vulnerable to undocumented confounders. Exclusion of patients who died or were discharged within 24 h, although methodologically necessary to analyze ICU variables, omits the most fulminant presentations and may underestimate any association between extreme hypoalbuminemia and ultra-early mortality. Being a single-center study further constrains generalizability; other institutions may use different fluid or albumin-replacement strategies, potentially yielding divergent albumin trajectories and outcome patterns. Moreover, detailed data on nutritional intake, timing, and dose of albumin infusions, diuretic use, and fluid balance were not recorded. Without these variables, we cannot determine whether correction of hypoalbuminemia or iatrogenic hyperalbuminemia was uniformly beneficial, neutral, or occasionally harmful. We also lacked systematic recording of inflammatory markers (e.g., CRP), organ dysfunction scores, and chronic comorbidities, preventing multivariable adjustment that might have uncovered a subtler independent effect of albumin. Finally, follow-up ended at hospital discharge; potential links between albumin trends and longer-term neurological or functional recovery remain unexplored.

These gaps shape a clear research agenda. Prospective, multicenter studies should confirm whether albumin truly lacks prognostic value when contemporary critical-care practices are applied, and whether this holds in resource-limited settings where hypoalbuminemia might persist. Such studies should collect granular data on nutrition, albumin administration, fluid strategy, and inflammatory burden, enabling adjustment for key confounders and exploration of effect modification. A randomized trial focused on targeted nutritional or albumin supplementation in hypoalbuminemic TBI patients could clarify whether proactively raising albumin influences intracranial dynamics, complication rates, or longer-term outcomes. Composite biomarker models, like combining albumin with lactate, CRP, leukocyte profiles, or neuro-imaging severity indices, also warrant evaluation, as emerging evidence suggests albumin’s predictive power improves when contextualized within broader physiologic stress indicators. In parallel, mechanistic work should investigate how albumin kinetics interact with blood-brain-barrier permeability, cerebral edema, drug binding, and immunomodulation, thereby illuminating whether albumin is purely a marker or occasionally a mediator of secondary brain injury. Lastly, incorporating 6 or 12-month functional outcomes and quality-of-life metrics will be essential to determine if albumin trends have prognostic relevance beyond the acute hospitalization window.

In summary, our study strengthens the case that, under modern intensive care, albumin concentration alone is an unreliable short-term prognosticator in severe TBI. Future investigations—prospective, mechanistically informed, and enriched with long-term follow-up—are needed to ascertain the contexts in which albumin becomes clinically actionable and to integrate it intelligently into holistic risk-stratification and therapeutic frameworks.

## 5. Conclusions

In this retrospective cohort of 925 patients with severe TBI, we observed a pronounced shift in serum albumin from predominantly normo- to hypoalbuminemia on admission to marked hyperalbuminemia at ICU discharge. Yet the albumin category at any measured time-point did not differentiate mortality, hospital or ICU length of stay, or ventilator-day burden. These findings indicate that, under contemporary neuro-critical-care protocols that routinely correct protein deficits, albumin concentration functions more as a transient marker of resuscitation dynamics rather than as an independent determinant of early clinical outcomes. Accordingly, while monitoring albumin remains essential for guiding fluid and nutritional therapy, it should not be relied upon as a stand-alone prognostic indicator in the acute phase of severe TBI. Future multicenter, prospective investigations incorporating detailed nutritional data, serial inflammatory biomarkers, and long-term functional endpoints are needed to determine whether albumin, alone or within composite indices, provides additional prognostic or therapeutic value beyond the hospital stay.

## Figures and Tables

**Table 1:** Baseline characteristics of patients with severe traumatic brain injury (TBI) included in the study.

	Observations	Percent	Cumulative Frequency	Cumulative Percent
Gender				
Female	219	23.68	219	23.68
Male	706	76.32	925	100.00
Race				
American Indian	1	0.11	1	0.11
Asian	137	14.81	138	14.92
Black	72	7.78	210	22.70
Native Hawaiian or Other Pacific Islander	2	0.22	212	22.92
Other	532	57.51	744	80.43
Unknown	17	1.84	761	82.27
White	164	17.73	925	100.00
Ethnicity				
Hispanic Origin	429	46.38	429	46.38
Non-Hispanic Origin	456	49.30	885	95.68
Unknown	40	4.32	925	100.00
Age				
0 < age < 15	17	1.84	17	1.84
15 ≤ age < 24	57	6.16	74	8.00
24 ≤ age ≤ 64	558	60.32	632	68.32
age > 65	293	31.68	925	100.00
Trauma Type				
Blunt	906	97.95	906	97.95
Penetrating	19	2.05	925	100.00
Mortality				
Non-survival	117	12.65	117	12.65
Survival	808	87.35	925	100.00

**Table 2:** Data of various ALBs at different time-points during hospitalization.

	Observations	Percent	Cumulative Frequency	Cumulative Percent
Albumin TA (Hospital Admission)				
Extreme Hyperalbuminemia	6	0.65	6	0.65
(≥5.5 g dL^−1^)				
Hyperalbuminemia	81	8.76	87	9.41
(4.7 to 5.4 g dL^−1^)				
Normoalbuminemia	601	64.97	688	74.38
(3.5 to 4.6 g dL^−1^)				
Hypoalbuminemia	233	25.19	921	99.57
(2.5 to 3.4 g dL^−1^)				
Extreme Hypoalbuminemia	4	0.43	925	100
(<2.5 g dL^−1^)				
Albumin RL1 (ICU Admission)				
Extreme Hyperalbuminemia	577	62.38	577	62.38
(≥5.5 g dL^−1^)				
Hyperalbuminemia	120	12.97	697	75.35
(4.7 to 5.4 g dL^−1^)				
Normoalbuminemia	210	22.7	907	98.05
(3.5 to 4.6 g dL^−1^)				
Hypoalbuminemia	18	1.95	925	100
(2.5 to 3.4 g dL^−1^)				
Extreme Hypoalbuminemia	0	0	925	100
(<2.5 g dL^−1^)				
Albumin RL2 (ICU Discharge)				
Extreme Hyperalbuminemia	578	62.59	578	62.59
(≥5.5 g dL^−1^)				
Hyperalbuminemia	146	15.78	724	78.37
(4.7 to 5.4 g dL^−1^)				
Normoalbuminemia	189	20.43	913	98.8
(3.5 to 4.6 g dL^−1^)				
Hypoalbuminemia	10	1.08	923	99.89
(2.5 to 3.4 g dL^−1^)				
Extreme Hypoalbuminemia	2	0.22	925	100
(<2.5 g dL^−1^)				
Albumin HD (Hospital Discharge)				
Extreme Hyperalbuminemia	33	3.57	33	3.57
(≥5.5 g dL^−1^)				
Hyperalbuminemia	202	21.84	235	25.41
(4.7 to 5.4 g dL^−1^)				
Normoalbuminemia	568	61.41	803	86.81
(3.5 to 4.6 g dL^−1^)				
Hypoalbuminemia	121	13.08	924	99.89
(2.5 to 3.4 g dL^−1^)				
Extreme Hypoalbuminemia	1	0.11	925	100
(<2.5 g dL^−1^)				
Albumin PD (Patient Death)				
Extreme Hyperalbuminemia	831	89.84	831	89.84
(≥5.5 g dL^−1^)				
Hyperalbuminemia	20	2.16	851	92
(4.7 to 5.4 g dL^−1^)				
Normoalbuminemia	58	6.27	909	98.27
(3.5 to 4.6 g dL^−1^)				
Hypoalbuminemia	16	1.73	925	100
(2.5 to 3.4 g dL^−1^)				
Extreme Hypoalbuminemia	0	0	925	100
(<2.5 g dL^−1^)				

**Table 3:** TA Albumin Category by Age Group.

Age Group	Extreme Hyper	Hyper	Normal	Hypo	Extreme Hypo	Total
<15 y	0	2	11	4	0	17
15–23 y	0	1	41	15	0	57
24–64 y	3	48	356	149	2	558
≥65 y	3	30	193	65	2	293
Total	6	81	601	233	4	925

**Table 4:** TA Albumin Category by Sex.

Sex	Extreme Hyper	Hyper	Normal	Hypo	Extreme Hypo	Total
Female	0	21	146	50	2	219
Male	6	60	455	183	2	706
Total	6	81	601	233	4	925

Albumin distributions were similar in men and women (χ^2^ = 4.38, *p* = 0.36).

**Table 5:** TA Albumin Category by Mechanism of Injury.

Mechanism	Extreme Hyper	Hyper	Normal	Hypo	Extreme Hypo	Total
Blunt	6	76	590	230	4	906
Penetrating	0	5	11	3	0	19
Total	6	81	601	233	4	925

Penetrating injuries were uncommon and displayed the same albumin profile as blunt injuries (χ^2^ = 7.87, *p* = 0.10).

**Table 6:** TA Albumin Category by AIS-Head Severity.

AIS-Head	Extreme Hyper	Hyper	Normal	Hypo	Extreme Hypo	Total
3	2	43	318	124	1	488
4	2	20	156	60	2	240
5	2	18	124	48	1	193
6	0	0	3	1	0	4
Total	6	81	601	233	4	925

Distribution of admission albumin categories across increasing AIS-Head severity scores. Albumin status did not vary significantly with anatomic head-injury severity (χ^2^ = 3.13, *p* = 0.99).

**Table 7:** TA Albumin Category vs. In-Hospital Mortality.

Albumin Category	Survivors (%)	Deaths (%)	Mortality Rate
Extreme hyperalbuminemia	6 (100%)	0 (0%)	0%
(≥5.5 g dL^−1^)			
Hyperalbuminemia	70 (86.4%)	11 (13.6%)	13.60%
(4.7–5.4 g dL^−1^)			
Normoalbuminemia	528 (87.9%)	73 (12.1%)	12.10%
(3.5–4.6 g dL^−1^)			
Hypoalbuminemia	200 (85.8%)	33 (14.2%)	14.20%
(2.5–3.4 g dL^−1^)			
Extreme hypoalbuminemia	4 (100%)	0 (0%)	0%
(<2.5 g dL^−1^)			
Total	808	117	12.70%

No significant difference in crude mortality across admission albumin strata (χ^2^ = 3.47, *p* = 0.32).

## Data Availability

Data for this study were requested from the Elmhurst Trauma registry and extracted using electronic medical records after receiving approval from the Institutional Review Board at our facility (Elmhurst Hospital Center).
